# Dynamic serum chloride trajectories predict short- and long-term mortality in patients with malignancies: a retrospective cohort study with external validation

**DOI:** 10.3389/fonc.2026.1814886

**Published:** 2026-09-29

**Authors:** Ruming Zhao, Shaojun Yang

**Affiliations:** Department of Oncology, Zibo Municipal Hospital, Zibo, Shandong, China

**Keywords:** group-based trajectory modeling, intensive care unit, malignant tumor, mortality, serum chloride trajectory

## Abstract

**Background:**

Hypochloremia is associated with poor prognosis in various malignant tumors. However, longitudinal trajectories of serum chloride in cancer patients and their relationship with mortality remain unexplored. This study aimed to delineate dynamic serum chloride trajectory patterns and analyze their association with mortality in patients with malignancies.

**Methods:**

This retrospective cohort study included 4,115 patients with malignant tumors from the MIMIC-IV database who had serial serum chloride measurements within the first 5 days of ICU admission, plus an additional 518 patients from Zibo Municipal Hospital for external validation. We applied group-based trajectory modeling (GBTM) to stratify patients into three distinct serum chloride trajectory classes. The primary outcomes were 28-day and 365-day all-cause mortality, which were assessed using Kaplan-Meier survival analysis and multivariable Cox proportional hazards regression. Subgroup and sensitivity analyses were further conducted to verify the robustness of the study findings.

**Results:**

Three distinct serum chloride trajectory patterns were identified in both the MIMIC-IV primary cohort and the external validation cohort: Class 1 (initial hyperchloremia resolving to normal levels), Class 2 (persistent normochloremia), and Class 3 (progressive hyperchloremia). In the fully adjusted Cox regression model (Model 3), Class 3 was an independent predictor of 28-day (HR = 1.55, 95% CI: 1.26-1.92) and 365-day all-cause mortality (HR = 1.38, 95%CI: 1.19-1.60) in the MIMIC-IV cohort. This prognostic value was validated in the external validation cohort, where Class 3 was also associated with a significantly increased 28-day mortality risk (HR = 1.90, 95%CI: 1.08-3.33). Kaplan-Meier survival analysis revealed that Class 3 patients had significantly lower survival rates at both 28-day and 365-day follow-ups. Subgroup analyses and sensitivity analyses further verified the robustness of the association between progressive hyperchloremia and adverse mortality outcomes.

**Conclusions:**

Progressive hyperchloremia trajectory independently predicts early and late mortality in cancer patients, and may offer a dynamic, low-cost biomarker for risk stratification.

## Introduction

Malignant tumors remain one of the foremost global public-health challenges, with incidence continuing to rise and imposing a heavy disease burden. In 2022, there were nearly 20 million new cancer cases and 9.7 million cancer-related deaths worldwide (1). In some developed countries and regions, malignant tumors have surpassed cardiovascular diseases as the leading cause of death (2). This high incidence and mortality not only inflict profound physical and psychological suffering on patients, markedly diminishing their quality of life, but also impose enormous economic costs on families and society, including soaring medical expenses, productivity losses, and long-term care expenditures (3, 4). Although recent advances in targeted therapy and immunotherapy have improved five-year survival rates for certain tumor types, there remains significant variability in survival time among cancer patients, with many experiencing rapid disease progression and subsequent mortality after diagnosis (5). Consequently, identifying readily accessible biomarkers that can be dynamically monitored and that influence malignant tumor prognosis is essential for more precise risk stratification and individualized management.

Serum chloride, the predominant extracellular anion, is indispensable for core physiological functions: maintaining membrane osmotic equilibrium, acid-base homeostasis, neuromuscular excitability, and gastric acid secretion during digestion (6). Accumulating evidence suggests that serum chloride is more than a routine electrolyte parameter; its derangement, particularly hypochloremia, is closely linked to the risk and prognosis of various diseases (7, 8). In heart failure, chronic kidney disease, and critical illness, hypochloremia has repeatedly emerged as an independent predictor of adverse outcomes (7, 9, 10). Previous studies have demonstrated that single-point hypochloremia is associated with poor prognosis in various malignant tumors (11–16). However, a solitary measurement captures only a fleeting snapshot and cannot depict the dynamic chloride trajectories that unfold over the disease course. Trajectory modelling integrates temporal data, evaluates cumulative risk, and tracks pattern evolution, thereby often outperforming static values in elucidating survival associations (17). To date, investigations into the longitudinal trajectories of serum chloride in patients with malignant tumors and their relationship with mortality remain uncharted.

Therefore, this study utilized clinical data from patients with malignancies from the Medical Information Mart for Intensive Care IV (MIMIC-IV) database and Zibo Municipal Hospital to delineate longitudinal trajectory patterns of serum chloride and analyze their association with all-cause mortality, thereby providing a potentially efficient and low-cost risk stratification tool for patients with malignancies.

## Methods

### Data source and participant selection

This retrospective observational cohort study integrated data from the MIMIC-IV database and the clinical data repository of Zibo Municipal Hospital, Shandong Province, China. The MIMIC-IV database is a public de-identified dataset containing 383,499 intensive care unit (ICU) admissions to Beth Israel Deaconess Medical Center (Harvard Medical School) from 2008 to 2019 (18). Author Ruming Zhao has completed the required ethical training for database access (ID: 71236130). Additionally, the study protocol was approved by the Institutional Review Board of Zibo Municipal Hospital (Ethical Approval No.: ZBMH202408), with informed consent waived due to the retrospective design and full anonymization of clinical data.

Study participants were screened from both cohorts. For the MIMIC-IV cohort: patients with a first ICU stay during their initial hospital admission and an ICD-9/10 diagnosis of malignant tumor were first identified (n = 8,416); those lacking serum chloride measurements within the first 5 ICU days were subsequently excluded (n = 4,301), yielding a final analytic cohort of 4,115 patients. For the Zibo Municipal Hospital cohort, eligible patients were those with pathologically confirmed malignant tumors admitted to the hospital from January 2023 to January 2025, with complete serum chloride measurements within the first 5 days and full clinical/follow-up data; patients with missing key indicators were excluded, resulting in 518 cases enrolled as the external validation cohort (**Figure 1**).

**Figure 1:**
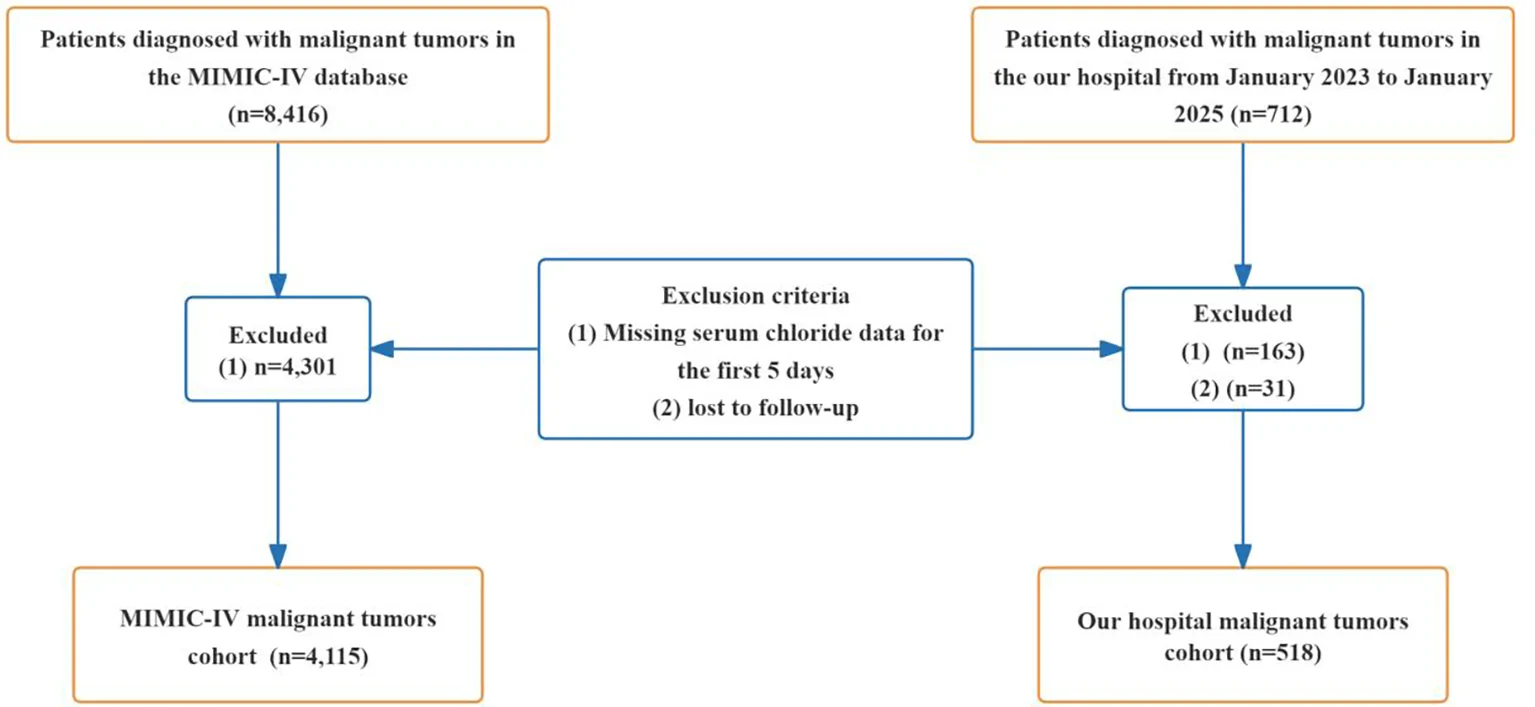
Flowchart of our participants.

### Variable selection

Guided by clinical relevance and statistical requirements, variables were selected across four domains: Demographics and vital signs: age, sex, weight, heart rate, systolic blood pressure (SBP), diastolic blood pressure (DBP), and respiratory rate. Laboratory indices: electrolytes (potassium, anion gap, bicarbonate), renal function markers [blood urea nitrogen (BUN), creatinine], calcium, glucose, platelets, and white blood cell count (WBC). Infusion data included all saline and crystalloid solutions administered over a period of 5 days. Illness-severity scores: Oxford Acute Severity of Illness score (OASIS), and Sequential Organ Failure Assessment (SOFA). Comorbidities: renal disease, chronic pulmonary disease, congestive heart failure, diabetes, hypertension, ischemic stroke, and myocardial infarction.

Variables with > 20% missing data were excluded; remaining missing values were imputed using random-forest multiple imputation.

### Exposure and outcome

We used group-based trajectory modelling (GBTM) guided by the Bayesian Information Criterion (BIC) to classify dynamic serum chloride patterns (19). Serial serum chloride measurement data from the first five days for patients in the MIMIC cohort and those treated in our hospital were entered into the GBTM to capture the dynamic trajectory of each patient. The optimal number of latent classes was selected according to the lowest BIC and the requirement that each trajectory comprise > 5% of individuals, balancing model fit and complexity (**Supplementary Tables 1**–**4**). The primary outcomes were 28-day and 365-day mortality.

### Statistical analysis

Continuous variables were expressed as mean ± standard deviation, and categorical variables as frequency (percentage). Between-group comparisons were performed using one-way ANOVA for continuous variables and the χ² test for categorical variables.

Kaplan-Meier curves with log-rank tests were used to compare survival across serum chloride trajectories. Subsequently, variables with baseline differences and clinical significance were incorporated into the multivariate Cox model, resulting in the construction of three stepwise adjustment models: Model 1 (unadjusted), Model 2 (adjusted for demographics), and Model 3 (fully adjusted for basic laboratory parameters, comorbidities, and disease-severity scores) were conducted to investigate the association between different serum chloride trajectories and mortality in patients with malignant tumors. Robustness of the findings was further assessed through subgroup and sensitivity analyses.

All analyses were conducted in R software (version 4.2.2), with statistical significance set at P < 0.05 (two-tailed).

## Results

### Serum chloride trajectories

Trajectory modeling analysis identified three distinct classes with significantly different dynamic patterns of serum chloride levels (**Table 1**).

**Table 1:** Serum chloride levels during the first 5 days of ICU stay.

Chloride levels	N	Day 1	Day 2	Day 3	Day 4	Day 5
MIMIC cohort						
Class 1	757	105.73 ± 5.44	104.31 ± 5.51	101.98 ± 5.59	99.75 ± 5.71	98.63 ± 5.04
Class 2	3009	101.65 ± 5.37	102.94 ± 5.58	103.09 ± 5.76	103.18 ± 5.48	103.14 ± 5.18
Class 3	349	95.31 ± 7.82	100.49 ± 7.74	103.68 ± 7.28	106.56 ± 7.17	108.00 ± 6.94
External cohort						
Class 1	40	106.07 ± 5.13	104.43 ± 5.67	102.39 ± 5.62	99.52 ± 6.62	98.60 ± 4.12
Class 2	432	101.92 ± 5.18	103.25 ± 5.52	103.38 ± 5.59	103.33 ± 5.13	103.48 ± 5.04
Class 3	46	95.89 ± 5.87	100.34 ± 6.40	102.91 ± 6.12	106.27 ± 5.43	108.30 ± 6.08

In the MIMIC cohort, Class 1 (hyperchloremia with declining trajectory) comprised 757 patients, Class 2 (normochloremia with stable trajectory) included 3,009 patients, and Class 3 (progressive hyperchloremia) included 349 patients. Class 1 decreased from 105.73 mmol/L to 98.63 mmol/L, Class 2 remained stable between 101.65 and 103.14 mmol/L, and Class 3 increased from 95.31 mmol/L to 108.00 mmol/L.

In the external validation cohort, the three subgroups comprised 40 (Class 1), 432 (Class 2), and 46 (Class 3) patients, respectively. Class 1 decreased from 106.07 mmol/L to 98.60 mmol/L, Class 2 remained stable between 101.92 and 103.48 mmol/L, and Class 3 increased from 95.89 mmol/L to 108.30 mmol/L.

**Figure 2** illustrates the trajectory of serum chloride level changes in the MIMIC database and the external validation cohort during the first five days. Latent class analysis identified three distinct patterns: Class 1-initial hyperchloremia followed by a gradual decline to normal levels; Class 2-stable normokalaemia throughout the observation period; and Class 3-progressive hyperchloremia with steadily rising chloride levels. These three categories accounted for 18.40%, 73.12%, and 8.48% of the MIMIC cohort, respectively, while their proportions in the external validation cohort were 7.72%, 83.40%, and 8.88%, respectively.

**Figure 2:**
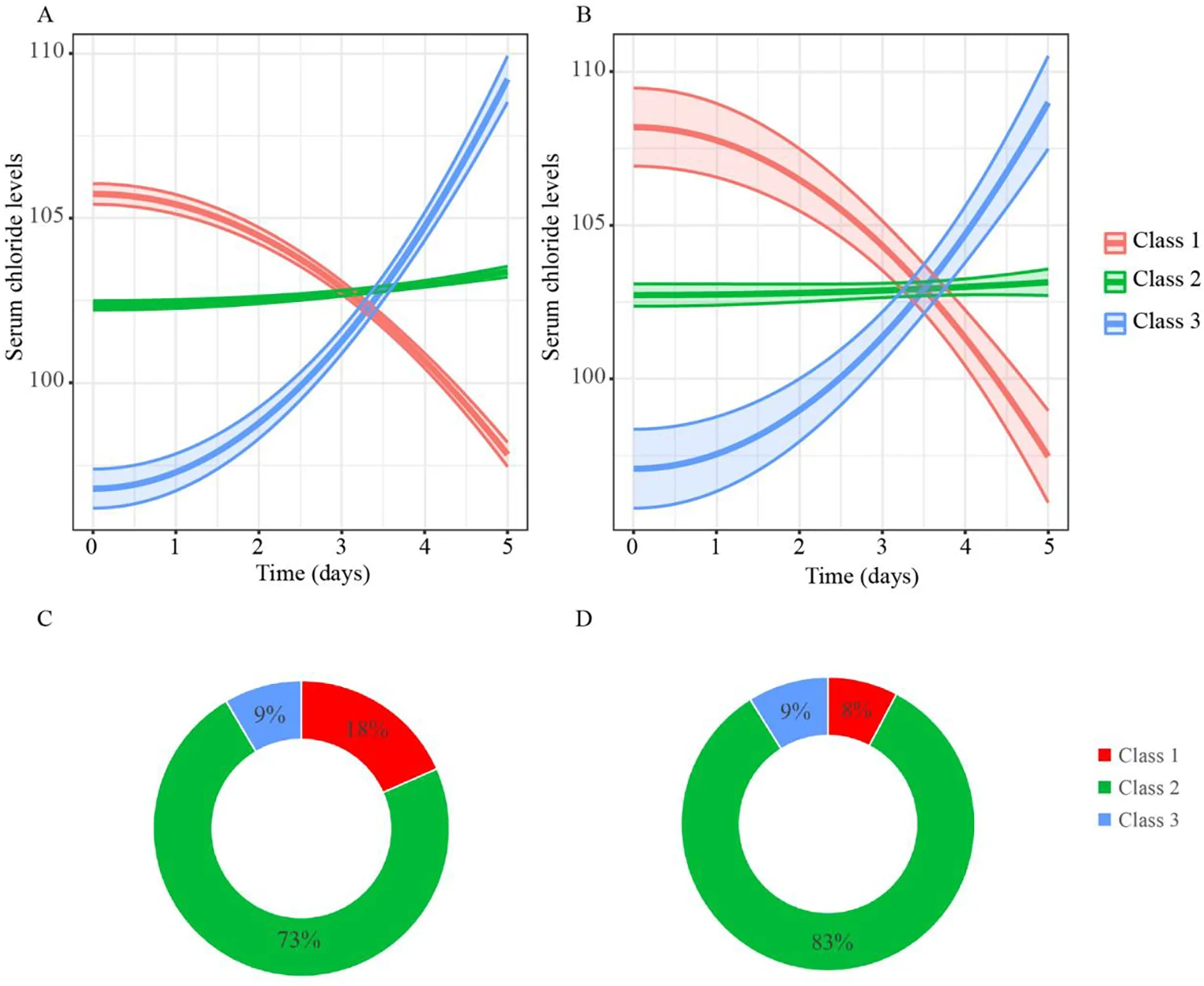
Serum chloride trajectory patterns in critically ill patients with malignant tumors during the first 5 days of ICU admission. Group-based trajectory modeling identified three distinct longitudinal patterns: Class 1 (initial hyperchloremia with a gradual decline to normal levels), Class 2 (persistent stable normokalaemia), and Class 3 (progressive hyperchloremia with a continuous upward trend). **(A)** represents the trajectory patterns derived from the MIMIC-IV database cohort; **(B)** represents the trajectory patterns derived from the external validation cohort (Zibo Municipal Hospital); **(C)** shows the percentages of three serum chloride trajectories in the MIMIC cohort. **(D)** shows the percentages of three serum chloride trajectories in the external validation cohort.

### Baseline characteristics of patients with malignancies

A total of 4,633 critically ill cancer patients were enrolled in this study, including 4,115 cases in the MIMIC-IV primary cohort and 518 in the Zibo Municipal Hospital external validation cohort (**Table 2**). The overall cohort had a mean age of 68.25 years, with a male predominance and a mean body weight of 79.18 kg. Inter-cohort comparisons showed no significant differences in baseline demographics (age, sex, weight), vital signs, most laboratory indices, comorbidities, and short-term mortality (all P>0.05), with only serum creatinine presenting a slight statistical difference between the two cohorts (P = 0.048).

**Table 2:** Comparison of characteristics in MIMIC and the external cohorts.

Characteristics	Total (n=4633)	MIMIC cohort (n=4115)	External cohort (n=518)	P
Age, years	68.25 ± 12.94	68.28 ± 12.97	68.00 ± 12.73	0.638
Male, n (%)	2749(59.34)	2445(59.42)	304(58.69)	0.786
Weight, Kg	79.18 ± 21.09	79.13 ± 21.14	79.60 ± 20.65	0.625
Heart rate, minute	73.93 ± 15.63	73.87 ± 15.63	74.37 ± 15.60	0.498
SBP, mmHg	89.74 ± 16.19	89.63 ± 16.27	90.65 ± 15.51	0.161
DBP, mmHg	46.64 ± 11.01	46.62 ± 11.07	46.82 ± 10.56	0.684
Respiratory rate, minute	12.45 ± 3.74	12.47 ± 3.72	12.32 ± 3.83	0.401
Platelet, K/µl	187.75 ± 120.63	188.22 ± 120.48	184.08 ± 121.92	0.467
WBC, K/µl	12.13 ± 16.96	12.17 ± 16.92	11.75 ± 17.28	0.594
Anion gap, mmol/L	12.70 ± 3.45	12.71 ± 3.49	12.58 ± 3.06	0.361
Bicarbonate, mmol/L	21.34 ± 4.75	21.33 ± 4.75	21.43 ± 4.72	0.640
BUN, mg/dl	24.41 ± 19.51	24.57 ± 19.85	23.18 ± 16.59	0.080
Calcium, mg/dl	8.08 ± 0.87	8.08 ± 0.88	8.08 ± 0.79	0.954
Creatinine, mg/dl	1.20 ± 1.14	1.21 ± 1.16	1.12 ± 0.88	0.048
Glucose, mg/dl	121.38 ± 40.18	121.26 ± 40.49	122.40 ± 37.69	0.518
Potassium, mmol/l	3.95 ± 0.57	3.95 ± 0.58	3.94 ± 0.55	0.759
OASIS	32.09 ± 8.41	32.08 ± 8.38	32.20 ± 8.66	0.751
SOFA	4.91 ± 3.41	4.90 ± 3.40	4.99 ± 3.50	0.589
Renal disease, n (%)	847(18.28)	768(18.66)	79(15.25)	0.067
Chronic pulmonary disease, n (%)	1215(26.22)	1074(26.10)	141(27.22)	0.622
Congestive heart failure, n (%)	1028(22.19)	917(22.28)	111(21.43)	0.700
Diabetes, n (%)	1207(26.05)	1072(26.05)	135(26.06)	1.000
Hypertension n, (%)	1936(41.79)	1712(41.60)	224(43.24)	0.506
Ischemic stroke, n (%)	159( 3.43)	143( 3.48)	16( 3.09)	0.744
Myocardial infarction, n (%)	583(12.58)	519(12.61)	64(12.36)	0.923
28-day mortality, n (%)	911(19.66)	816(19.83)	95(18.34)	0.456

SBP, systolic blood pressure; DBP, diastolic blood pressure; WBC, white blood cell count; BUN, blood urea nitrogen; SOFA, Sequential Organ Failure Assessment.

**Supplementary Table 5** presents the baseline characteristics of patients with malignancies in the MIMIC cohort, stratified by trajectory class. A total of 4,115 patients were included in the MIMIC cohort; the overall mean age was 68.28 years, and 59.42% were male. Respiratory rate differed significantly across classes, with Class 3 having the highest value. Laboratory findings revealed multiple significant differences among classes. In Class 3, the anion gap, serum urea nitrogen, creatinine, and total fluid volume infused within the preceding 5 days were all significantly elevated. OASIS scores and SOFA scores were significantly higher in Class 3, indicating greater organ dysfunction in this group. Regarding comorbidities, ischemic stroke was significantly more prevalent in Class 3. Mortality outcomes varied dramatically across trajectory classes. The 28-day and 365-day mortality rates were significantly higher in Class 3.

**Supplementary Table 6** presents the baseline characteristics of patients with malignancies in the external cohort, stratified by trajectory class. The overall mean age was 68 years, with 58.69% being male. Respiratory rate was highest in Class 3. Laboratory findings revealed significant differences in anion gap and bicarbonate levels, with Class 3 showing the highest anion gap and Class 2 demonstrating the highest bicarbonate. Both OASIS and SOFA scores were significantly higher in Class 1 and Class 3 compared to Class 2, indicating greater illness severity in these trajectory groups. Regarding comorbidities, congestive heart failure was significantly more prevalent in Class 1 compared to Class 2 and Class 3. Notably, 28-day mortality varied significantly across trajectory classes, with Class 3 exhibiting the highest mortality rate, approximately double that of the other two classes.

### Kaplan-Meier analysis

Kaplan-Meier survival analysis revealed significant differences in all-cause survival across the three serum chloride trajectory classes (log-rank test, P < 0.05; **Figure 3**). Class 3 showed the poorest survival outcomes at 28 days and 365 days of follow-up in the MIMIC-IV cohort, and this poor prognostic trend was consistent in the 28-day survival of the external validation cohort; by contrast, Classes 1 and 2 consistently maintained markedly higher survival probabilities across all follow-up periods and cohorts.

**Figure 3:**
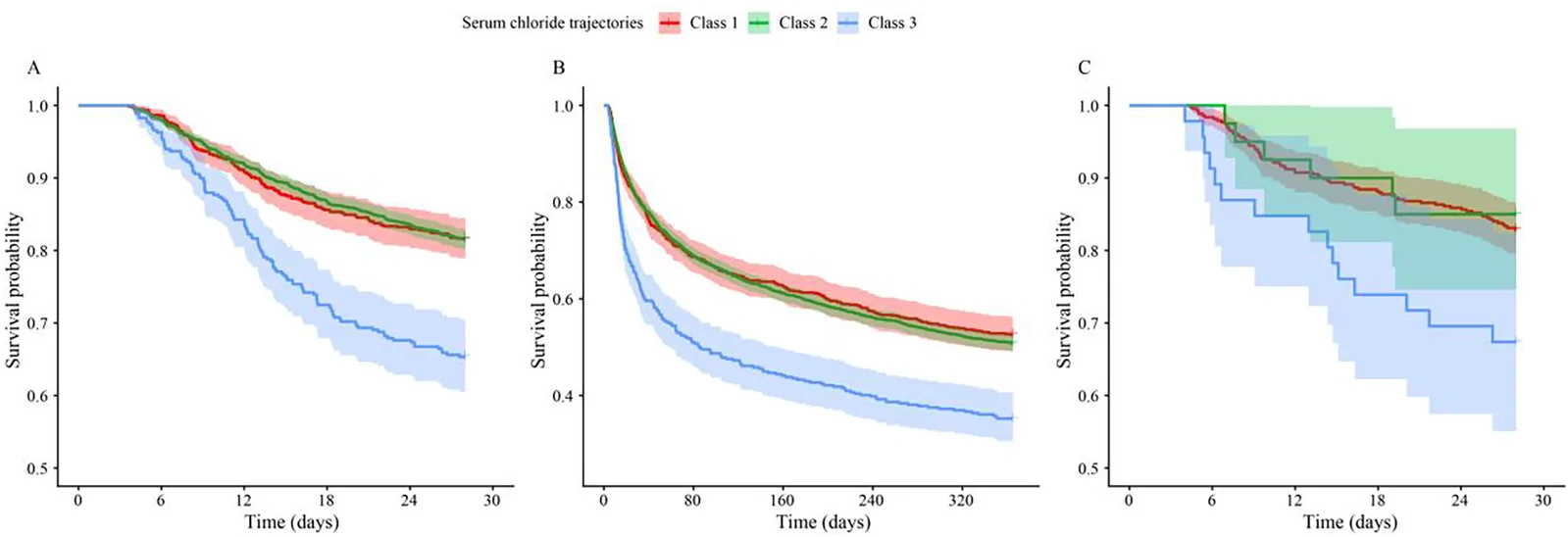
Kaplan-Meier curves for all-cause mortality in critically ill cancer patients by serum chloride trajectories. **(A)** 28-day mortality (MIMIC cohort); **(B)** 365-day mortality (MIMIC cohort); **(C)** 28-day mortality (external cohort). Survival probabilities differed significantly across the three classes (Log-rank test, all P <0.05).

### Multivariable Cox regression

We further evaluated the prognostic value of serum chloride trajectories for mortality in patients with malignant tumors using three sequentially adjusted Cox regression models, with analyses conducted for both the MIMIC-IV cohort and the external validation cohort (**Table 3**). For the MIMIC-IV cohort, in the unadjusted model (Model 1), Class 3 was associated with a 2.10-fold (95%CI: 1.73-2.56) higher risk of 28-day mortality and a 1.65-fold (95%CI: 1.44-1.90) higher risk of 365-day mortality compared with Class 2 (the reference group). Even after full adjustment for vital signs, laboratory indices, disease severity scores, and comorbidities (Model 3), the increased mortality risk in Class 3 persisted and remained significant (28-day HR = 1.55; 365-day HR = 1.38). For the external validation cohort, consistent prognostic trends were observed for 28-day mortality: Class 3 was linked to a significantly elevated mortality risk in the unadjusted model (HR = 2.14, 95%CI: 1.23-3.73), with the association remaining significant after full multivariable adjustment (Model 3: HR = 1.90, 95%CI: 1.08-3.33). Notably, in both cohorts, Class 1 exhibited no statistically significant difference in mortality risk relative to Class 2 across all three models (P > 0.05 for all comparisons).

**Table 3:** Relationship between serum chloride trajectories and mortality in patients with malignant tumors in different models.

Chloride levels	Model 1		Model 2		Model 3	
	HR (95% CI)	P	HR (95% CI)	P	HR (95% CI)	P
28-day mortality in MIMIC cohort						
Class						
2	ref		ref		ref	
1	1.00 (0.83,1.21)	0.994	1.03 (0.85,1.24)	0.769	1.02 (0.84,1.24)	0.848
3	2.10 (1.73,2.56)	<.001	2.11 (1.72,2.58)	<.001	1.55 (1.26,1.92)	<.001
365-day mortality in MIMIC cohort						
Class						
2	ref		ref		ref	
1	0.96 (0.86,1.08)	0.518	0.97 (0.86,1.09)	0.557	0.96 (0.85,1.08)	0.514
3	1.65 (1.44,1.90)	<.001	1.66 (1.44,1.91)	<.001	1.38 (1.19,1.60)	<.001
28-day mortality in external cohort						
Class						
2	ref		ref		ref	
1	0.87 (0.38,2.00)	0.744	0.83 (0.36,1.92)	0.671	0.81 (0.35,1.87)	0.623
3	2.14 (1.23,3.73)	0.007	2.17 (1.23,3.80)	0.007	1.90 (1.08,3.33)	0.025

HR, Hazard Ratio, CI, Confidence Interval.

Model 1: Crude.

Model 2: Adjusted for age, sex, and weight.

Model 3: Adjusted for age, sex, weight, heart rate, SBP, respiratory rate, WBC, anion gap, bicarbonate, BUN, creatinine, glucose, potassium, OASIS, and SOFA, renal disease, chronic pulmonary disease, congestive heart failure, ischemic stroke, and myocardial infarction (MIMIC cohort); Adjusted for age, sex, weight, respiratory rate, BUN, fluid infusion, and ischemic stroke (External cohort).

### Subgroup analyses

Due to the limited number of patients in the external validation cohort, subgroup analysis was conducted exclusively in the MIMIC cohort. Subgroup analyses showed that the association between serum chloride trajectory and mortality risk remained consistent across different populations in the MIMIC cohort (**Figures 4**, **5**). In every subgroup examined, Class 3 remained linked to increased risks of both 28-day and 365-day mortality. Notably, an age-related interaction was detected for 28-day mortality among patients with malignant tumors (P for interaction < 0.05), such that the association between Class 3 and mortality was markedly stronger in those younger than 70 years than in those aged 70 years or older.

**Figure 4:**
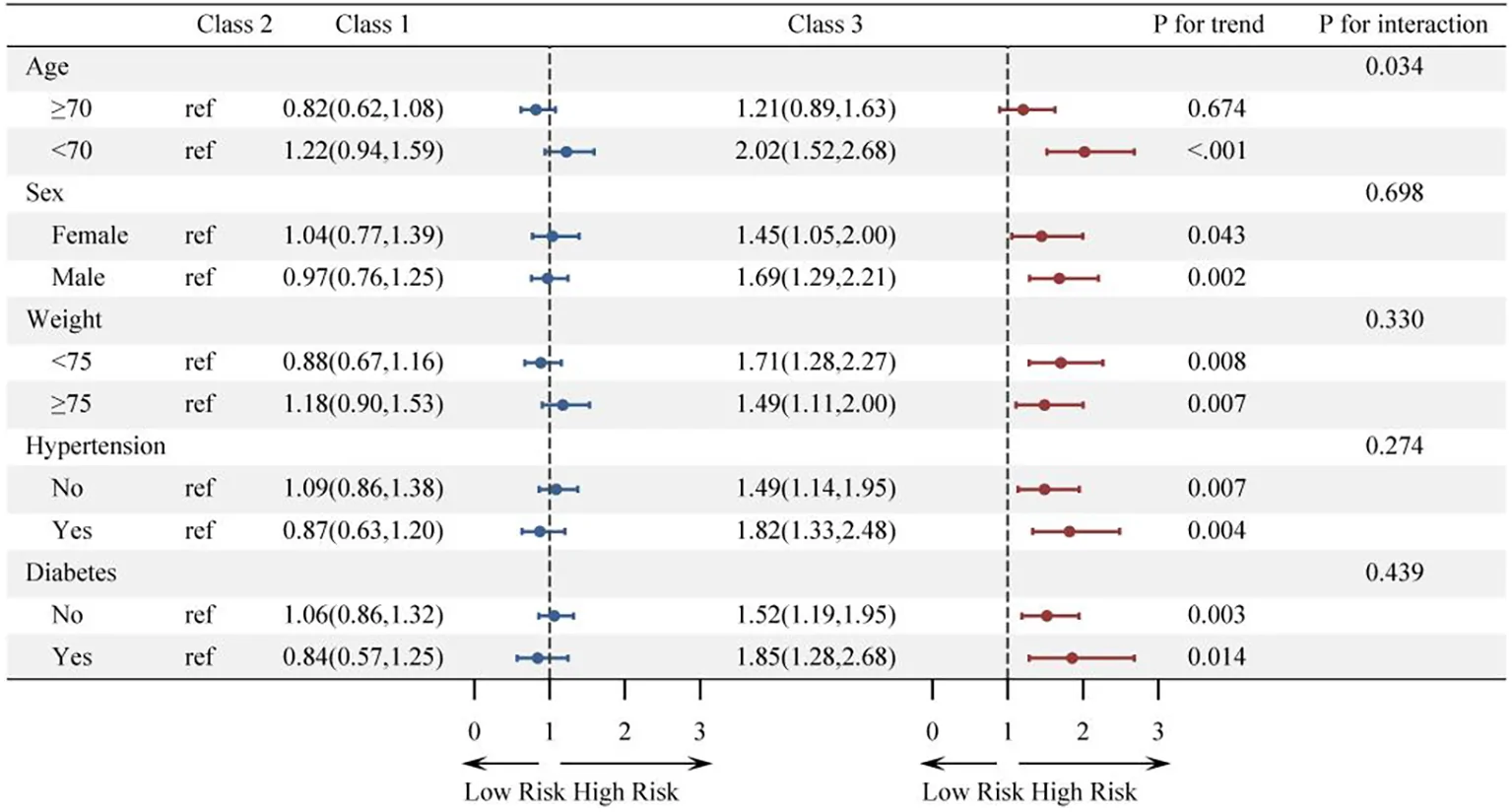
Subgroup analysis for the relationship between serum chloride trajectories and 28-day mortality in patients with malignant tumors in MIMIC cohort.

**Figure 5:**
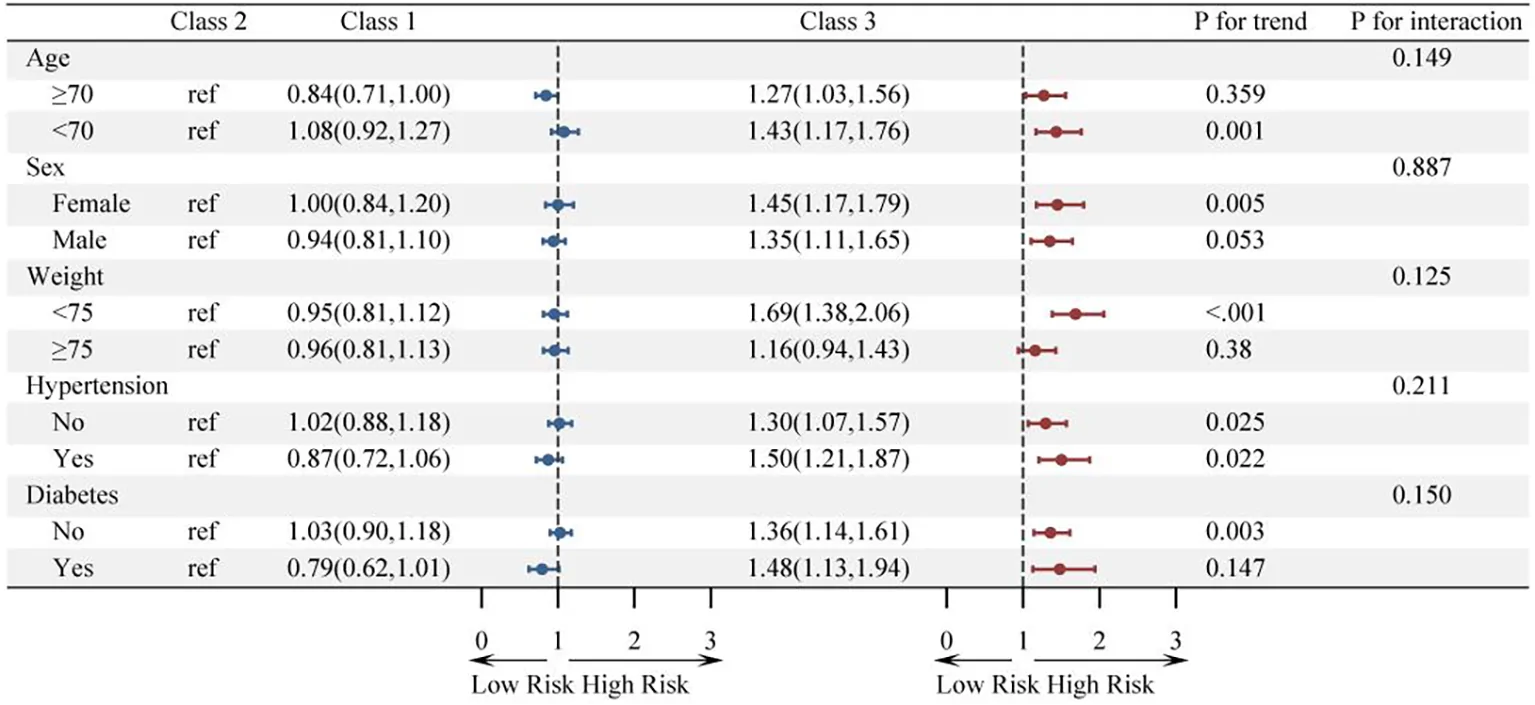
Subgroup analysis for the relationship between serum chloride trajectories and 365-day mortality in patients with malignant tumors in the MIMIC cohort.

### Sensitivity analyses

Sensitivity analyses for the MIMIC cohort in which patients with congestive heart failure were excluded confirmed that Class 3 remained independently associated with increased 28-day and 365-day mortality (Model 3: 28-day HR = 1.82, 95%CI: 1.43-2.31; 365-day HR = 1.48, 95%CI: 1.25-1.75) (**Supplementary Table 7**). Similar findings were observed when patients with myocardial infarction were additionally excluded, with Class 3 still showing significantly higher mortality at both 28 days and 365 days (**Supplementary Table 8**).

## Discussion

This study is the first to use longitudinal trajectory modelling in critically ill patients with malignancies to delineate three distinct serum chloride evolution patterns—initial hyperchloremia resolving to normal levels (Class 1), stable normochloremia (Class 2), and progressive hyperchloremia (Class 3)—and to demonstrate that the progressive hyperchloremia trajectory is independently associated with significantly higher risks of 28-day and 365-day all-cause mortality. These findings transcend the constraints of conventional single-point electrolyte assessments and provide a dynamic prognostic tool for risk stratification in this high-risk population.

Over the past few years, trajectory modelling has gained considerable traction in critical-care research. For instance, Bhavani et al. applied GBTM to serial temperature measurements collected during the first 72 h of ICU stay in 12,413 patients with sepsis and identified four distinct phenotypes: rapid defervescence hyperthermia, slow defervescence hyperthermia, normothermia, and hypothermia (20). Hypothermic patients exhibited the highest mortality (9.5%), whereas those with rapid defervescence hyperthermia had the lowest (2.9%). Importantly, this classification was externally validated in a cohort of 19,053 patients, underscoring temperature trajectories as a biomarker of sepsis heterogeneity and outcome. Similarly, Jiang et al. analyzed C-reactive protein (CRP) trajectories in 1,464 septic patients and found that the persistently high-CRP pattern was associated with an in-hospital mortality of 32.6% (OR = 2.19; 95%CI: 1.55, 3.11), whereas the moderate-CRP pattern had the lowest mortality (18.1%), confirming that dynamic CRP changes constitute an independent predictor of death (21). Trajectory approaches have also been validated in acute ischemic stroke. A new prospective study continuously monitored heart rate for 72 h in 224 patients after mechanical thrombectomy. Latent-class mixture modelling revealed five heart-rate trajectories; the “extremely high-rate” group (6.3%) had an 18-fold increased risk of poor 3-month functional outcome (OR = 18.32; 95%CI: 2.20, 95.52), while the low-rate group had the best prognosis (22). Collectively, these studies demonstrate that longitudinal trajectory analysis can furnish critical information for early risk stratification, prognostic assessment, and individualized intervention. Building on this evidence, we are the first to delineate three distinct serum chloride trajectories in critically ill patients with malignancy and to show that progressive hyperchloremia (Class 3) is independently associated with increased risks of both early and late all-cause mortality, while initial hyperchloremia that resolves to normal levels (Class 1) shows no significant prognostic difference from stable normochloremia (Class 2).

This study found that the mortality at 28 days and 365 days was significantly increased in the progressive hyperchloremia group (low serum chloride levels at admission followed by sustained elevation over 5 days) than that in the stable serum chloride group. This finding does not contradict previous reports linking admission hypochloremia to poor prognosis in various malignancies, as the progressive hyperchloremia group also exhibited hypochloremia at admission, indicating that these patients were already in a severe pathophysiological state upon ICU admission. The association between progressive hyperchloremia and increased mortality may be attributed to two factors. First, patients in this group may have received more aggressive saline resuscitation due to their more severe pathophysiological state, while the hyperchloremic properties of 0.9% sodium chloride solution (154 mmol/L) can lead to hyperchloremic metabolic acidosis (23). Second, the gradual increase in chloride ions following admission hypochloremia, accompanied by significant elevations in blood urea nitrogen and creatinine, reflects the progression of acute kidney injury, which is an independent risk factor for poor prognosis in malignant tumor patients (24–26).

Compared with single-point measurements, trajectory analysis offers two core advantages: (1) The same absolute chloride value (e.g., 110 mmol/L) carries markedly different prognoses depending on trajectory, exposing the limitations of static indices; and (2) a dynamic intervention target, analogous to fluid-management trajectory studies, chloride trajectories can inform the timing of therapeutic actions rather than relying solely on endpoint values.

Despite employing advanced trajectory modeling to analyze serum chloride dynamics in a large cohort of critically ill cancer patients, this study has several limitations. First, the retrospective design only included patients with repeated chloride measurements, potentially excluding those with rapid deterioration or early discharge. Second, although we adjusted for covariates including SOFA scores and common clinical indicators, the lack of tumor-specific variables (such as chemotherapy regimens and TNM staging) prevented us from fully accounting for treatment heterogeneity or the influence of the tumor microenvironment on the results. Finally, although we demonstrated that progressive hyperchloremia is independently associated with increased mortality across the overall cohort of critically ill patients with malignancies, we were unable to perform reliable subgroup analyses stratified by specific tumor types due to insufficient sample sizes within individual categories. Future multicenter studies with larger, tumor-specific cohorts are needed to validate the generalizability of our findings across diverse malignancies. These limitations highlight the need for multicenter prospective studies incorporating tumor genomics and standardized monitoring protocols to validate our findings.

## Conclusion

This is the first study to delineate 5-day serum chloride trajectories in patients with malignant tumors and demonstrates that progressive hyperchloremia independently increases 28-day and 365-day mortality. Dynamic chloride ion monitoring may enable early identification of high-risk individuals, providing a potentially low-cost tool for precise risk stratification and targeted interventions.

## Data Availability

The raw data supporting the conclusions of this article will be made available by the authors, without undue reservation.
