# Supplementary material for: Dynamic serum chloride trajectories predict short- and long-term mortality in patients with malignancies: a retrospective cohort study with external validation
Source: Front Oncol. 2026 Sep 29;16:1814886. doi: 10.3389/fonc.2026.1814886 (PMC13657915; doi:10.3389/fonc.2026.1814886)
Supplement: Supplementary file 1 [file SupplementaryFile1.docx]

**Supplementary Materials**

**Supplementary Table 1. Parameters of different trajectory models for serum chloride in MIMIC cohort.**

**Supplementary Table 2. Posterior probabilities in the three-trajectory model in MIMIC cohort.**

**Supplementary Table 3. Parameters of different trajectory models for serum chloride in external cohort.**

**Supplementary Table 4. Posterior probabilities in the three-trajectory model in external cohort.**

**Supplementary Table 5. Baseline characteristics of patients with malignancies in MIMIC cohort.**

**Supplementary Table 6. Baseline characteristics of patients with malignancies in external cohort.**

**Supplementary Table 7. Relationship between serum chloride trajectories and mortality after excluding patients with congestive heart failure in MIMIC cohort.**

**Supplementary Table 8. Relationship between serum chloride trajectories and mortality after excluding patients with myocardial infarction in MIMIC cohort.**

| **Supplementary Table 1. Parameters of different trajectory models for serum chloride in MIMIC cohort** | | | | | | | |
| --- | --- | --- | --- | --- | --- | --- | --- |
| number of grouping | BIC | Loglik | Class 1 | Class 2 | Class 3 | Class 4 | Class 5 |
| 1 | 116748.56 | -58357.64 | 100 |  |  |  |  |
| 2 | 114846.03 | -57393.89 | 76.35 | 23.65 |  |  |  |
| 3 | 114056.44 | -56986.61 | 18.40 | 73.12 | 8.48 |  |  |
| 4 | 113864.59 | -56878.20 | 9.33 | 20.85 | 67.12 | 2.70 |  |
| 5 | 113836.58 | -56851.71 | 2.07 | 11.54 | 20.97 | 63.50 | 1.92 |
| Abbreviation: BIC, Bayesian Information Criterion; Loglik, Log-likelihood. | | | | | | | |

| **Supplementary Table 2. Posterior probabilities in the three-trajectory model in MIMIC cohort** | | | |
| --- | --- | --- | --- |
| Chloride trajectories | Probabilities 1 | Probabilities 2 | Probabilities 3 |
| Class 1 | 0.82 | 0.18 | 0.00 |
| Class 2 | 0.08 | 0.89 | 0.03 |
| Class 3 | 0.00 | 0.12 | 0.88 |

| **Supplementary Table 3. Parameters of different trajectory models for serum chloride in external cohort** | | | | | | | |
| --- | --- | --- | --- | --- | --- | --- | --- |
| number of grouping | BIC | Loglik | Class 1 | Class 2 | Class 3 | Class 4 | Class 5 |
| 1 | 14651.09 | -7313.05 | 100.00 |  |  |  |  |
| 2 | 14498.04 | -7227.15 | 74.52 | 25.48 |  |  |  |
| 3 | 14412.11 | -7174.80 | 7.72 | 83.40 | 8.88 |  |  |
| 4 | 14410.47 | -7164.61 | 3.86 | 53.47 | 38.80 | 3.86 |  |
| 5 | 14426.95 | -7163.48 | 2.90 | 6.18 | 60.62 | 27.80 | 2.51 |
| Abbreviation: BIC, Bayesian Information Criterion; ng, number of grouping; Loglik, Log-likelihood. | | | | | | | |

| **Supplementary Table 4. Posterior probabilities in the three-trajectory model in external cohort** | | | |
| --- | --- | --- | --- |
| Chloride trajectories | Probabilities 1 | Probabilities 2 | Probabilities 3 |
| Class 1 | 0.89 | 0.09 | 0.03 |
| Class 2 | 0.21 | 0.79 | 0.00 |
| Class 3 | 0.15 | 0.00 | 0.85 |

z

| **Supplementary Table 5. Baseline characteristics of patients with malignancies in MIMIC cohort** | | | | | |
| --- | --- | --- | --- | --- | --- |
| Characteristics | Total (n=4,115) | Class 1 (n=757) | Class 2 (n=3,009) | Class 3 (n=349) | P |
|  |  |  |  |  |  |
| Age, years | 68.28 ± 12.97 | 67.69 ± 13.50 | 68.38 ± 12.84 | 68.67 ± 12.88 | 0.355 |
| Male n (%) | 2445 (59.42) | 442 (58.39) | 1809 (60.12) | 194 (55.59) | 0.215 |
| Weight, Kg | 79.13 ± 21.14 | 78.07 ± 20.51 | 79.50 ± 21.13 | 78.16 ± 22.49 | 0.166 |
| Heart rate, minute | 73.87 ± 15.63 | 73.69 ± 15.18 | 73.80 ± 15.74 | 74.93 ± 15.69 | 0.415 |
| SBP, mmHg | 89.63 ± 16.27 | 89.01 ± 15.42 | 89.86 ± 16.37 | 88.98 ± 17.10 | 0.324 |
| DBP, mmHg | 46.62 ± 11.07 | 45.73 ± 10.70 | 46.92 ± 11.10 | 45.97 ± 11.49 | 0.016 |
| Respiratory rate, minute | 12.47 ± 3.72 | 12.08 ± 3.63 | 12.48 ± 3.72 | 13.16 ± 3.84 | <.001 |
| Platelet, K/µl | 188.22 ± 120.48 | 182.26 ± 114.83 | 190.65 ± 120.06 | 180.11 ± 134.79 | 0.097 |
| WBC, K/µl | 12.17 ± 16.92 | 12.43 ± 18.03 | 11.95 ± 16.71 | 13.52 ± 16.24 | 0.235 |
| Anion gap, mmol/L | 12.71 ± 3.49 | 12.33 ± 3.24 | 12.65 ± 3.42 | 14.07 ± 4.25 | <.001 |
| Bicarbonate, mmol/L | 21.33 ± 4.75 | 20.86 ± 4.52 | 21.58 ± 4.67 | 20.18 ± 5.65 | <.001 |
| BUN, mg/dl | 24.57 ± 19.85 | 24.33 ± 21.27 | 23.82 ± 18.63 | 31.47 ± 24.95 | <.001 |
| Calcium, mg/dl | 8.08 ± 0.88 | 8.02 ± 0.79 | 8.10 ± 0.88 | 8.00 ± 1.02 | 0.013 |
| Creatinine, mg/dl | 1.21 ± 1.16 | 1.18 ± 1.19 | 1.19 ± 1.15 | 1.41 ± 1.17 | 0.003 |
| Glucose, mg/dl | 121.26 ± 40.49 | 121.42 ± 35.13 | 121.14 ± 40.27 | 121.88 ± 51.74 | 0.942 |
| Potassium, mmol/l | 3.95 ± 0.58 | 3.96 ± 0.57 | 3.95 ± 0.56 | 3.88 ± 0.69 | 0.089 |
| OASIS | 32.08 ± 8.38 | 32.60 ± 8.34 | 31.77 ± 8.31 | 33.61 ± 8.84 | <.001 |
| Fluid infusion, mL | 3836.48 ± 4152.60 | 3761.87 ± 3924.32 | 3757.62 ± 4066.87 | 4678.21 ± 5163.68 | <.001 |
| SOFA | 4.90 ± 3.40 | 4.86 ± 3.28 | 4.74 ± 3.34 | 6.33 ± 3.81 | <.001 |
| Renal disease, n (%) | 768 (18.66) | 124 (16.38) | 583 (19.38) | 61 (17.48) | 0.140 |
| Chronic pulmonary disease, n (%) | 1074 (26.10) | 208 (27.48) | 781 (25.96) | 85 (24.36) | 0.515 |
| Congestive heart failure, n (%) | 917 (22.28) | 175 (23.12) | 668 (22.20) | 74 (21.20) | 0.759 |
| Diabetes, n (%) | 1072 (26.05) | 189 (24.97) | 782 (25.99) | 101 (28.94) | 0.372 |
| Hypertension n, (%) | 1712 (41.60) | 307 (40.55) | 1254 (41.67) | 151 (43.27) | 0.689 |
| Ischemic stroke, n (%) | 143 (3.48) | 31 (4.10) | 93 (3.09) | 19 (5.44) | 0.044 |
| Myocardial infarction, n (%) | 519 (12.61) | 102 (13.47) | 383 (12.73) | 34 (9.74) | 0.206 |
| 28-day mortality, n (%) | 816 (19.83) | 139 (18.36) | 556 (18.48) | 121 (34.67) | <.001 |
| 365-day mortality, n (%) | 2065 (50.18) | 359 (47.42) | 1480 (49.19) | 226 (64.76) | <.001 |
| Abbreviation: SBP, systolic blood pressure; DBP, diastolic blood pressure; WBC, white blood cell; BUN, blood urea nitrogen; OASIS, oxford acute severity of illness score; SOFA, sequential organ failure assessment. | | | | | |

| **Supplementary Table 6. Baseline characteristics of patients with malignancies in external cohort** | | | | | |
| --- | --- | --- | --- | --- | --- |
| Characteristics | Total (n=518) | Class 1 (n=40) | Class 2 (n=432) | Class 3 (n=46) | P |
|  |  |  |  |  |  |
| Age, years | 68.00 ± 12.73 | 67.32 ± 13.97 | 67.73 ± 12.71 | 71.10 ± 11.63 | 0.220 |
| Male n (%) | 304(58.69) | 24(60.00) | 256(59.26) | 24(52.17) | 0.640 |
| Weight, Kg | 79.60 ± 20.65 | 75.30 ± 15.74 | 79.81 ± 20.44 | 81.39 ± 25.79 | 0.347 |
| Heart rate, minute | 74.37 ± 15.60 | 78.50 ± 14.55 | 73.80 ± 15.59 | 76.09 ± 16.22 | 0.140 |
| SBP, mmHg | 90.65 ± 15.51 | 85.04 ± 16.79 | 91.57 ± 15.31 | 86.91 ± 14.96 | <.001 |
| DBP, mmHg | 46.82 ± 10.56 | 44.94 ± 10.16 | 47.18 ± 10.71 | 45.04 ± 9.25 | 0.214 |
| Respiratory rate, minute | 12.32 ± 3.83 | 12.58 ± 3.44 | 12.15 ± 3.82 | 13.67 ± 4.07 | 0.033 |
| Platelet, K/µl | 184.08 ± 121.92 | 163.55 ± 75.29 | 187.42 ± 124.97 | 170.63 ± 125.02 | 0.365 |
| WBC, K/µl | 11.75 ± 17.28 | 8.99 ± 4.72 | 11.74 ± 16.40 | 14.18 ± 28.66 | 0.382 |
| Anion gap, mmol/L | 12.58 ± 3.06 | 11.05 ± 2.70 | 12.59 ± 2.83 | 13.80 ± 4.60 | <.001 |
| Bicarbonate, mmol/L | 21.43 ± 4.72 | 19.45 ± 4.75 | 21.75 ± 4.60 | 20.13 ± 5.29 | <.001 |
| BUN, mg/dl | 23.18 ± 16.59 | 21.35 ± 16.70 | 22.96 ± 16.26 | 26.87 ± 19.29 | 0.242 |
| Calcium, mg/dl | 8.08 ± 0.79 | 7.78 ± 0.74 | 8.11 ± 0.78 | 8.03 ± 0.88 | 0.040 |
| Creatinine, mg/dl | 1.12 ± 0.88 | 0.98 ± 0.58 | 1.12 ± 0.89 | 1.31 ± 0.99 | 0.199 |
| Glucose, mg/dl | 122.40 ± 37.69 | 115.78 ± 35.06 | 123.69 ± 37.41 | 116.07 ± 41.91 | 0.219 |
| Potassium, mmol/l | 3.94 ± 0.55 | 3.82 ± 0.54 | 3.94 ± 0.54 | 4.04 ± 0.63 | 0.175 |
| OASIS | 32.20 ± 8.66 | 35.80 ± 10.27 | 31.55 ± 8.25 | 35.24 ± 9.62 | <.001 |
| SOFA | 4.99 ± 3.50 | 6.05 ± 3.83 | 4.71 ± 3.34 | 6.72 ± 4.10 | <.001 |
| Renal disease, n (%) | 79(15.25) | 3(7.50) | 71(16.44) | 5(10.87) | 0.222 |
| Chronic pulmonary disease, n (%) | 141(27.22) | 9(22.50) | 122(28.24) | 10(21.74) | 0.503 |
| Congestive heart failure, n (%) | 111(21.43) | 15(37.50) | 86(19.91) | 10(21.74) | 0.035 |
| Diabetes, n (%) | 135(26.06) | 7(17.50) | 113(26.16) | 15(32.61) | 0.280 |
| Hypertension n, (%) | 224(43.24) | 15(37.50) | 184(42.59) | 25(54.35) | 0.232 |
| Ischemic stroke, n (%) | 16(3.09) | 1(2.50) | 14(3.24) | 1(2.17) | 0.901 |
| Myocardial infarction, n (%) | 64(12.36) | 4(10.00) | 55(12.73) | 5(10.87) | 0.837 |
| 28-day mortality, n (%) | 95(18.34) | 6(15.00) | 74(17.13) | 15(32.61) | 0.031 |
| Abbreviation: SBP, systolic blood pressure; DBP, diastolic blood pressure; WBC, white blood cell count; BUN, blood urea nitrogen; SOFA, Sequential Organ Failure Assessment. | | | | | |

| **Supplementary Table 7. Relationship between serum chloride trajectories and mortality after excluding patients with congestive heart failure in MIMIC cohort** | | | | | | |
| --- | --- | --- | --- | --- | --- | --- |
| Chloride levels | Model 1 | | Model 2 | | Model 3 | |
|  | HR (95%CI) | P | HR (95%CI) | P | HR (95%CI) | P |
| 28-day mortality | | | | | | |
| Class |  |  |  |  |  |  |
| 2 | ref |  | ref |  | ref |  |
| 1 | 1.06 (0.85,1.32) | 0.597 | 1.08 (0.86,1.35) | 0.509 | 1.08 (0.86,1.36) | 0.487 |
| 3 | 2.42 (1.94,3.03) | <.001 | 2.47 (1.96,3.10) | <.001 | 1.82 (1.43,2.31) | <.001 |
| 365-day mortality | | | | | | |
| Class |  |  |  |  |  |  |
| 2 | ref |  | ref |  | ref |  |
| 1 | 0.99 (0.87,1.13) | 0.895 | 0.98 (0.86,1.12) | 0.778 | 0.97 (0.84,1.12) | 0.684 |
| 3 | 1.78 (1.51,2.08) | <.001 | 1.81 (1.54,2.14) | <.001 | 1.48 (1.25,1.75) | <.001 |
| HR, Hazard Ratio; CI, Confidence Interval.  Model 1: Crude.  Model 2: Adjusted for age, sex, and weight.  Model 3: Adjusted for age, sex, weight, heart rate, SBP, respiratory rate, WBC, anion gap, bicarbonate, BUN, creatinine, glucose, potassium, fluid infusion, OASIS, and SOFA, renal disease, chronic pulmonary disease, ischemic stroke, and myocardial infarction. | | | | | | |

| **Supplementary Table 8. Relationship between serum chloride trajectories and mortality after excluding patients with myocardial infarction in MIMIC cohort** | | | | | | |
| --- | --- | --- | --- | --- | --- | --- |
| Chloride levels | Model 1 | | Model 2 | | Model 3 | |
|  | HR (95%CI) | P | HR (95%CI) | P | HR (95%CI) | P |
| 28-day mortality | | | | | | |
| Class |  |  |  |  |  |  |
| 2 | ref |  | ref |  | ref |  |
| 1 | 1.09 (0.89,1.33) | 0.389 | 1.11 (0.91,1.36) | 0.312 | 1.13 (0.92,1.39) | 0.238 |
| 3 | 2.14 (1.73,2.65) | <.001 | 2.13 (1.71,2.65) | <.001 | 1.60 (1.28,2.01) | <.001 |
| 365-day mortality | | | | | | |
| Class |  |  |  |  |  |  |
| 2 | ref |  | ref |  | ref |  |
| 1 | 1.01 (0.89,1.14) | 0.931 | 1.01 (0.89,1.14) | 0.927 | 1.02 (0.90,1.16) | 0.787 |
| 3 | 1.64 (1.42,1.91) | <.001 | 1.66 (1.42,1.93) | <.001 | 1.39 (1.18,1.62) | <.001 |
| HR, Hazard Ratio; CI, Confidence Interval.  Model 1: Crude.  Model 2: Adjusted for age, sex, and weight.  Model 3: Adjusted for age, sex, weight, heart rate, SBP, respiratory rate, WBC, anion gap, bicarbonate, BUN, creatinine, glucose, potassium, fluid infusion, OASIS, and SOFA, renal disease, chronic pulmonary disease, congestive heart failure, and ischemic stroke. | | | | | | |
